# Effects of heat stress on reproduction and gene expression in sheep

**DOI:** 10.1590/1984-3143-AR2024-0067

**Published:** 2025-03-17

**Authors:** Galma Boneya Arero, Ozge Ozmen

**Affiliations:** 1 Ambo University, College of Agriculture and Veterinary Medicine, School of Veterinary Medicine, Department of Veterinary Laboratory Technology, Ambo, Ethiopia; 2 Ankara University, Graduate School of Health Science, Faculty of Veterinary Medicine, Department of Veterinary Genetics, Ankara, Turkey

**Keywords:** heat stress, reproduction, adaptation, genes, sheep

## Abstract

Small ruminant farming plays a pivotal role in agriculture, especially in developing countries due to sheep's diverse functions and capacity to acclimate to varying temperatures. This review comprehensively explored the impact of rising temperatures on reproductive processes, reproductive function encoding gene expression, and sheep's ability to adapt to heat stress. Several mechanisms contribute to sheep's resilience to heat stress, encompassing morphological, behavioral, physiological, and genetic adaptations. It has been shown that heat stress compromises fertility by affecting follicular development, ovulation rate, estrous behavior, rates of conception, embryonic survival, and fetal development, while also disrupting sperm production and motility, and increasing the incidence of structurally abnormal sperm in males. Estimates suggested that heat stress may reduce conception rates from 20% to 27%. Essential genes encoding the Gonadotrophin-releasing hormone, Follicle-stimulating hormone receptor, Luteinizing hormone receptor, Estradiol receptor, progesterone receptor, and Inhibin play a critical role in elucidating how heat stress impacts the reproductive performance of sheep. Furthermore, the resilience of sheep in facing heat stress adversities is associated with a specific heat shock factor. When an animal is under heat stress, Heat shock factors get activated and stimulate the production of Heat Shock Proteins (HSPs). Emphasis should be given to identifying specific genes and candidate genes that confer protection against heat stress and conducting comprehensive research to unravel how sheep adapt to demanding local climatic conditions to enhance production and profitability, improve animal welfare, and for genetic conservation and breeding programs.

## Introduction

Sheep farming occupies a pivotal position in the agricultural sector, especially in developing nations, due to the myriad advantages sheep provide. These benefits encompass the production of milk, meat, skin, wool, and supplementary sources of income, as well as their inherent resilience to varying climatic conditions (Rathwa et al., 2017). Their exceptional resilience in thriving under extreme heat and cold conditions underscores their importance in the global agricultural sector (Wojtas et al., 2014). Over centuries, sheep have undergone physical and genetic adaptations to contend with changing environments (Sejian et al., 2018). Animals like sheep employ various mechanisms to survive severe weather conditions. Thermoregulation encompasses adaptive strategies such as morphological, behavioral, physiological, blood biochemistry, neuro-endocrine, cellular, and molecular adjustments that are crucial for animals' survival in specific habitats (Das et al., 2016). The comprehension of thermoregulation extends beyond genetic regulation or physical trait modifications. Epigenetics assumes significance as it responds to environmental stressors without altering the DNA sequence, thereby playing a pivotal role in preserving the stabilization of the genome, organization of nuclear cells, transcriptional processes, and imprinting (Bartels et al., 2018).

Despite their high tolerance to harsh conditions, these animals often face challenges in their well-being, production, and reproduction due to environmental stress. This leads to significant economic losses each year, especially in tropical, subtropical, and arid regions (van Wettere et al., 2021). Global sheep meat production reaches nearly 9 million tonnes annually, with developing countries leading the way (Mazinani and Rude, 2020). In terms of consumption, sheep meat ranks fourth, following pork, poultry, and beef. Additionally, about 20.8% of global dairy products come from sheep and goats, contributing 1.3% and 1.9% to the world’s total milk production, respectively. However, it's estimated that 2.1 million potential lambs are lost due to heat stress (days > 32 °C), costing the sheep industry $97 million annually in Australia (Mazinani and Rude, 2020). These encompass diminished rates of production and reproduction, impeded growth, reduced quantity and quality of milk, and compromised natural immunity. Consequently, animals under heat stress are more susceptible to diseases (Amitha et al., 2019). The physiological effects of heat stress on sheep include increased respiration rate, increased sweating rate, increased body temperature, increased heart rate, panting, reduced feed intake, disruptions in water, protein, energy, and mineral metabolism, changes in the secretion of hormones and metabolism of blood, and compromised reproductive functions due to the redirection of energy resources towards vital life processes (Sejian et al., 2011)

In reproduction, the harmful effects of heat stress have been extensively recognized across various species, particularly in dairy cattle, buffaloes, goats, and sheep. A previous study has shown that heat stress can reduce conception rates by 20% to 27% (Scholtz et al., 2013). Moreover, heightened temperatures affect gonadotropin release, leading to decreased production of estrogen and progesterone (Scholtz et al., 2013). Numerous research has underscored the harmful impacts of heat stress on follicular dynamics, consequently diminishing reproductive capabilities in cattle (Wolfenson et al., 2000). These consequences encompass a lower percentage of estrus exhibition, shortened estrus duration, alterations in the length of the estrus cycle, decreased conception and lambing rates, as well as reduced birth weights of lambs (Maurya et al., 2004). Furthermore, studies have shown that heat stress can significantly inhibit the response of developing follicles to Follicle-Stimulating Hormone (FSH) secretion (Kiyma et al., 2004) and cause the degeneration of sperm (Marai et al., 2007). Heat Stress (HS) can also induce germ cell apoptosis, leading to DNA damage and disrupting the normal maturation of sperm (Hamilton et al., 2018), thereby compromising endometrial functions and secretory processes in goats (Amitha et al., 2019).

Exposure to thermal stress at the molecular level leads to a range of abnormalities in cell function. These abnormalities encompass modifications in biological molecules, disruptions in cellular processes, alterations in metabolic reactions, the initiation of oxidative cellular damage, and the activation of pathways that can result in both apoptosis and necrosis. The outcome, whether resulting in the cell's survival, acclimatization, or death of the cell, depends on the timing and effectiveness of these changes (Archana et al., 2017). Extensive research has been conducted on Heat Shock Proteins (HSPs) as genes affected by heat shock. Recent research indicates that thermal stress not only induces HSPs but also influences numerous other genes (Archana et al., 2017). Some of these genes respond to various stressors, revealing a broad response of the cell to stress, while others may be specific to certain types of stress. Researchers are particularly interested in exploring genes or potential genes associated with the ability to withstand high temperatures, a characteristic shaped by natural selection in species residing at lower altitudes (Kaushik et al., 2016). Although numerous research studies have focused on sheep and their reproductive traits under heat stress conditions, primarily through conducting physical observations, there is a noticeable gap in research regarding its effects on genetic traits. The exact molecular mechanisms that explain how heat stress affects genetic traits associated with animal reproduction are still largely unknown. Therefore, investigating the fundamental mechanisms of changes induced due to heat stress in reproductive traits and genes in sheep is crucial. The main objectives of this review are to highlight the alterations in reproduction and gene expression induced by heat stress, to enhance our comprehension of how sheep acclimate to heat stress conditions and to identify specific genes, including potential candidates, that are responsible for heat resilience in sheep.

## Mechanisms of adaptation to heat stress in sheep

**Figure 1:**
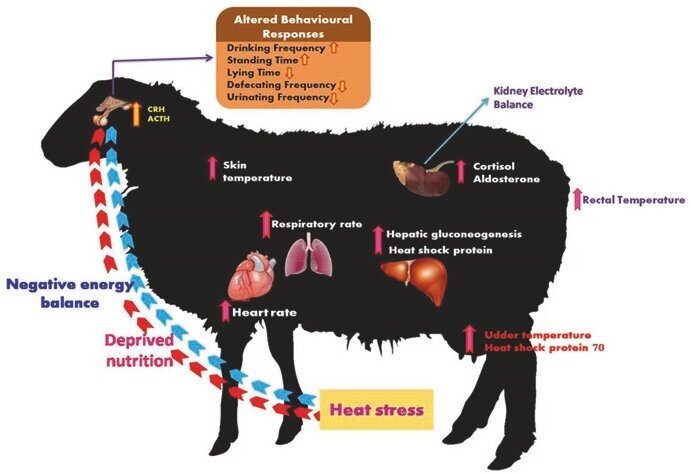
Pictorial representation of various organs and the corresponding events associated with sheep adaptation (Sejian et al., 2017a).

Sheep display adaptive responses to diverse environmental conditions that can be influenced by various factors such as climate, food resources, wool density, and disease susceptibility (Sejian et al., 2017b). Compared to other ruminant species, sheep are more resilient to heat stress (Bakheit et al., 2017). The severity of Heat Stress (HS) in sheep is typically assessed using the Temperature-Humidity Index (THI). Generally, sheep begin to experience heat stress at a THI above 72 (López et al., 2015). However, other studies on heat-tolerant breeds shown that HS symptoms may not appear until the THI reaches 82 units, with stress categorized into three levels: moderate (82–83.9), severe (84–85.9), and very severe (≥86) (Marai et al., 2007). According to Koluman et al. (2016), THI 70 or lower values are considered as comfortable, 75–78 is stressful, and values higher than 78 are distressful because animals are unable to maintain thermoregulatory mechanisms or normal body temperature. Regarding Temperature, the thermoneutral zone for most sheep ranges between 12°C and 27°C, meaning they remain comfortable and maintain normal body functions within this temperature range (Marai et al., 2008; Sejian et al., 2017a). However, for tropical sheep breeds, the upper threshold extends to 30°C, reflecting their enhanced natural tolerance for warmer climates (Neves et al., 2009).​ Another study reported that tropical sheep breeds are comfortable in environmental temperatures as high as 38 ◦C, while temperate breeds perform better within a temperature range of 5–25 ◦C (Gandhi and Arjava Sharma, 2016). The ability to withstand and adapt to heat stress differs among individuals and breeds. Tropical sheep breeds are more highly adapted to arid and semi-arid regions, having efficient thermoregulatory mechanisms (Sejian et al., 2010a). A previous study has shown that DNA analysis to identify genes linked to adaptive traits of Chokla, Magra, Marwari, and Madras Red sheep breeds is investigated as being heat-tolerant in Indian sheep breeds (Singh et al., 2016). Additionally, Awassi (Gootwine, 2011) and Omani (Mahgoub et al., 2010) sheep are among the carpet-type wool, light-colored fleece, thinner skin, shorter hairs, and fatter tails have better heat dissipation in hot environments. In regions characterized by high temperatures, sheep face challenges such as heat stress and scarcity of food resources, which require them to travel longer distances to access limited grazing lands during adverse weather conditions. Sheep possess remarkable capabilities to endure various temperature ranges and effectively convert the forage which is low in quality into high-quality animal protein (Shinde and Sejian, 2013). They employ a myriad of behavioral, morphological, physiological, and genetic mechanisms to proficiently endure heat stress. These mechanisms constitute pivotal adaptations that enable them to effectively manage elevated temperatures (de La Salles et al., 2020). As shown in Figure 1.

## Behavioral adaptations

One of the most rapid and impactful mechanisms of adaptation observed in sheep when exposed to heat is their behavioral response. These responses include searching for shade (Solórzano-Montilla et al., 2018), minimizing feed intake (Attia, 2016), decreasing activity, choosing cooler feeding times during the day (Singh et al., 2017), standing closer to each other, increasing water intake and drinking frequency (Rana et al., 2014) and reducing urine production (Chedid et al., 2014). In contrast, a study conducted by (Shilja et al., 2016) revealed that Indigenous sheep breeds exhibited limited variations in standing time, lying time, and urination frequency in response to summer heat stress, indicating their adaptation to high-temperature conditions.

## Morphological adaptations

Sheep display a variety of morphological adaptations, encompassing body size, type of hair and tail, skin color, and coat (Chedid et al., 2014). Indigenous breeds that have evolved in tropical and subtropical regions have a higher adaptive capacity to heat stress than exotic breeds. They manifest unique physical traits such as fleece akin to carpet, light-colored wool, thin dermis, short fur, and a fatty tail, all of which facilitate effective heat dissipation in warmer climates. The color of the fleece significantly influences the sheep's capacity to adapt to heat stress. In hot tropical regions, light or white-coated sheep are favored as they reflect 50% to 60% of direct sunlight, unlike their dark-coated counterparts (McManus et al., 2009).

According to research conducted by Singh et al. (2016), smaller sheep with dark skin and white hair and those with lengthy, slender limbs, demonstrate superior heat stress tolerance. Dark or brown-coated sheep are less suited to elevated temperatures compared to their white-coated counterparts. Despite smaller animals presenting a larger specific surface area, rendering them more susceptible to solar radiation, they exhibit enhanced heat tolerance. Seixas et al. (2017) validated this observation by contrasting a large breed (Santa Ines) with a smaller breed (Morada Nova) of hair sheep in terms of heat tolerance. Animals sporting dark, lengthy, and dense coats are at risk of overheating, while those with thinner coats and short, dense hairs facilitate efficient heat dissipation, allowing for improved air circulation and heat transfer (Seixas et al., 2017). This highlights the superiority of hair sheep over wool sheep in hot climates, although wool sheep also exhibit heat tolerance in similar conditions (McManus et al., 2020). Furthermore, sheep with fat tails exhibit adaptability in challenging conditions, particularly during droughts and food shortages, as the fat tail enhances heat transfer (Zhang et al., 2021).

## Physiological adaptations

Animals employ various physiological adaptation mechanisms to mitigate heat stress (de La Salles et al., 2020). Key indicators such as alterations in Heart rate (HR), Respiratory rate (RR), and Rectal temperature (RT) are crucial in elucidating the physiological adaptation process of small ruminants (Kaushik et al., 2016). Numerous studies have demonstrated that the rate of respiration and the temperature taken from the rectum serve as effective markers for thermal stress, aiding in assessing the severity of heat conditions (Berihulay et al., 2019). In their study, Gupta et al., (2013) reported a significant rise in rectal temperature and respiratory rate. The rectal temperature increased from 38.97°C to 39.35°C, while the respiratory rate increased from 43.66 breaths per minute to 77.33 breaths per minute. Similarly, the study conducted by Hooda and Upadhyay (2014) revealed significant increments in Respiratory Rate (RR) and Body Temperature (BT) at temperatures of 40, 42, and 44 ◦C, as the subject was subjected to higher thermal conditions. Ewes exposed to stressors such as heat, nutritional deficiencies, and physical exertion display heightened physiological indicators, including rectal temperature, pulse rate, respiratory rate, and sweating rate, as part of their homeostatic response (Sejian et al., 2012b). The increase in respiratory rate helps the dissipation of body heat (Slimen et al., 2019).

Moreover, it has been observed that the group exposed to multiple stressors exhibits a significantly higher Skin Temperature (ST) and scrotal temperature. Various studies suggest that these parameters could be valuable biomarkers for assessing Malpura rams' adaptive capacity. Specifically, scrotal temperature has been identified as a more reliable indicator in comparison to skin temperature when evaluating the effects of various stressors (Sejian et al., 2017b). The differences in temperatures between the scrotum and the skin can be explained by the fact that wool covers the body but not the scrotum. Additionally, the scrotum serves an important function as a thermoregulatory organ in sheep (Sejian et al., 2017b).

İn the heat-stressed environments, breeds originating from temperate and arid climates exhibited elevated rectal temperatures and respiratory rates, whereas those from tropical regions displayed lower values. Supporting this argument, Romero et al. (2013) conducted a study to evaluate the thermoregulatory ability of specific breeds residing in temperate and tropical areas under heat-stress conditions. İn Romero et al. (2013) study, Pelibuey, and Suffolk sheep were compared to their capacity to regulate body temperature under environmental hyperthermia by measuring their differences in the cellular response to Heat Stress (HS). In the experiment, Pelibuey (native to tropical) and Suffolk (native to temperate) were kept in a climatic chamber for 6 h daily for 10 days (temperatures within the 18 to 39.5 °C range). As chamber temperature rose, sheep rectal temperature increased in both groups, but to a lesser extent in Pelibuey (0.3 °C) than in Suffolk sheep (0.7 °C). Rana et al. (2014) examined the impact of heat stress on blood parameters in sheep by dividing the animals into three groups based on exposure duration: zero hours (T0), four hours (T4), and eight hours (T8) of direct sunlight. The study found that Red Blood Cell (RBC) count, Hemoglobin Concentration (Hb%), and Packed Cell Volume (PCV%) increased significantly with rising heat stress. The study also emphasized that temperature stress led to noticeable alterations in specific blood parameters among domestic sheep.

## Genetic adaptations

Genetic adaptation is a phenomenon that involves the heritable characteristics displayed by animals, which play a crucial role in the survival and expansion of populations (Niyas et al., 2015). These adaptive traits generally demonstrate low heritability. Species thriving in specific environments possess distinctive adaptive attributes determined by genetic factors (de La Salles et al., 2020). The progress in molecular biotechnologies has introduced new avenues for investigating gene expression and identifying cells' essential responses to heat stress (Renaudeau et al., 2012). Gupta et al. (2013) studied that *HSP32, HSP40, HSP60, HSP70, HSP90, HSP110,* and many other genes are found to be increased during hyperthermic stress and responsible for cellular thermo-tolerance by changing their pattern of expression. These genes are crucial for cellular adaptation and are considered potential biomarkers for understanding stress adaptation mechanisms in sheep (Collier et al., 2012). Exposure to heat stress has been shown to increase the expression of several genes, including *Heat Shock Protein* (*HSP)* genes, *Apoptotic* genes, as well as various *Cytokines and Toll-like receptor* genes. Several studies have provided evidence supporting the upregulation of *HSP70* as a crucial molecular marker for assessing the response to heat stress in small ruminants (Renaudeau et al., 2012; Gupta et al., 2013; Shilja et al., 2016) Moreover, several other genes have been identified to be associated with heat tolerance in sheep and goats, including superoxide dismutase, nitric oxide synthase, thyroid hormone receptor, and prolactin receptor (Renaudeau et al., 2012).

Heat stress induces the activation of Heat Shock Factor-1 (HSF1), thereby enabling the upregulation of *Heat Shock Proteins (HSP) genes*. Based on their molecular weights, these proteins are categorized into different families (Sejian et al., 2010b). Notably, extensively studied proteins with approximate molecular weights of 90, 70, and 27 kDa are commonly referred to as *Heat Shock Proteins HSP90, HSP70, and HSP27*, respectively (Collier et al., 2008). A study conducted by Singh et al., (2017) explored various sheep breeds and revealed that genotypes adapted to favorable conditions exhibited reduced expression of three *Heat shock Protein genes (HSP90AA1, HSPA1A, and HSPA8)*. Furthermore, certain breeds of sheep, known for their high-quality wool, demonstrated enhanced regulation of body temperature and cellular viability when exposed to heat stress, potentially through a mechanism associated with *HSP70* (Maurya et al., 2010).

In a study conducted by Shilja et al. (2016), it was discovered that the adrenal gland of sheep exhibited elevated levels of Messenger RNA (mRNA) for *Heat Shock Protein 70 (HSP70)* when exposed to various stressors. This increase in expression is regarded as part of the adaptive response to stress induced by heat and nutrition. Interestingly, the researchers observed a notable increase in adrenal *HSP70* expression in sheep subjected to multiple stressors, in contrast to those experiencing heat stress (Lima et al., 2014). This distinction could be attributed to the additional nutritional stress encountered by the group with multiple stressors (Shilja et al., 2016). The heightened expression of *HSP70* in the adrenal gland may also be associated with increased activity of the adrenal cortex in cortisol production, as indicated by the study by Shilja et al. (2016) Furthermore, the plasma levels of *HSP70* and the pattern of *HSP70* expression in peripheral blood mononuclear cells followed a comparable pattern, with significantly higher values observed in individuals exposed to multiple stressors compared to both the control group and those facing individual stressors (heat or nutrition) (Shilja et al., 2016).

## Effects of heat stress on sheep reproduction

Sheep exposed to ambient temperatures above 30°C suffer from heat stress, which negatively impacts their productivity and reproductive performance. Prolonged heat exposure disrupts vital physiological processes, reducing growth rates, milk production, fertility, and lambing success, ultimately threatening the sustainability of the production system (De et al., 2020). Confronting severe weather conditions prompts animals to deploy compensatory and adaptive measures to maintain internal stability crucial for survival. However, acclimating to these conditions diverts their energy towards these adaptive processes, affecting their reproductive capacities (Indu et al., 2014). Heat stress in animals impacts fertility through two primary pathways (Kumar et al., 2018). Firstly, it directly elevates body temperatures, influencing the reproductive system. Secondly, it activates the Hypothalamic-pituitary-adrenal axis, influencing reproductive functions (Indu et al., 2014). Even a slight rise in body temperature due to environmental heat stress can disturb the reproductive processes of both female and male sheep. This heat-induced hyperthermia affects the endocrine system, leading to modifications within the hypothalamic-hypophyseal-gonadal axis, thereby impairing reproductive functions such as gamete formation, embryo development, and placenta formation (Rossi, 2017). Research has demonstrated that exposure to heat stress is likely to significantly raise abnormal sperm count and decrease the viability of sperm (Khan et al., 2020). Furthermore, heat stress can also induce apoptosis in germ cells, cause DNA damage, and disrupt normal sperm maturation (Hamilton et al., 2018).

In various mammals, research has demonstrated that glucocorticoids can impact reproductive function at the hypothalamic-pituitary level through a variety of mechanisms (Lima et al., 2014). Activation of the Hypothalamic-Pituitary-Adrenal (HPA) axis has been shown to indirectly influence the synthesis of gonadotrophin-releasing hormone and the Luitinizing hormone preovulatory surge by downregulating the expression of kisspeptin (Khan et al., 2020). Kisspeptin is widely acknowledged for its pivotal role in initiating pubertal development and regulating ovarian cycles. In females, the inhibition of kisspeptin in specific hypothalamic regions can disturb ovarian cyclicity and impede the onset of puberty before adulthood (Luo et al., 2016). A comprehensive meta-analysis has indicated that heat stress can diminish oestrus duration, prolong cycle intervals, enhance embryonic mortality, and reduce fetal birth weight in sheep (Pérez et al., 2020; Zhang et al., 2021).

## Effects of heat stress on ewes

The efficacy of female reproductive processes hinges upon the appropriate development of follicles in the ovaries, ovulation occurring promptly, and the production of hormones (van Wettere et al., 2021). Endocrine interactions regulate these coordinated processes, which can be disrupted under stressful conditions (Dobson et al., 2012). It significantly influences the development of follicles and oocytes by modulating the secretion and patterns of progesterone, luteinizing hormone, and follicle-stimulating hormone throughout the estrous cycle (Ozawa et al., 2005). Heat stress negatively impacts germ cells, early embryos, and other reproductive cells before fertilization. One study in sheep found that exposing oocytes to elevated temperatures (41.8°C) for 12 hours reduced their ability to complete nuclear maturation and post-fertilization development (Kumar et al., 2017). A recent study found that all stages of growing preantral follicles, including primary and secondary follicles, were vulnerable to the harmful effects of heat stress in vitro in cattle (Aguiar et al., 2020). Additionally, heat stress can change the biochemical environment of follicles, indirectly affecting granulosa cells and the developmental potential of oocytes (Webb et al., 2002). Oocytes play a crucial role in regulating the development and function of granulosa cells from follicular organization to ovulation. This mutual interaction between oocytes and surrounding somatic cells throughout follicle development and maturation is likely sensitive to the effects of heat stress. As a direct effect, heat stress in female sheep diminishes fertility by impacting ovulation, estrus expression, conception rates, embryonic survival, and fetal development. Some experimental studies have shown that heat stress, whether applied before or during behavioral estrus, significantly increases the occurrence of cytoplasmic vacuoles, globules, ruptured Oolemma, cracks in the zona pellucida, fertilization failure, and early embryonic loss (Kleemann and Walker, 2005; Marai et al., 2008). Field studies using environmental chambers showed that heat stress during the peri-estrous period leads to increased fertilization failure and early embryo loss (Kleemann and Walker, 2005). The likelihood of ewes returning to estrus, indicating fertilization failure, rose by 3.44% for each additional day with temperatures of 32°C or higher during the mating period. Elevated temperatures following mating also seemed to contribute to greater embryo loss (Kleemann and Walker, 2005).

The early embryo is highly sensitive to elevated temperatures, displaying biphasic thermosensitivity. It remains particularly vulnerable until the early morula stage, after which its ability to tolerate heat gradually improves (Pöhland et al., 2020). During the initial stages of development, the activation of the embryonic genome triggers increased transcription and protein synthesis. At this point, several Heat Shock Proteins (HSPs) and antioxidant enzymes are overexpressed, enhancing the molecular defense mechanisms that support thermotolerance. Numerous in vivo and in vitro studies have demonstrated that Heat Stress (HS) exposure in oocytes and early embryos does not prevent their progression to the blastocyst stage (Stamperna et al., 2021; Dovolou et al., 2023).

Insufficient food intake, metabolic stress, weakened immune function, irregular breeding cycles, and reduced estrus behavior due to high temperatures indirectly harm conception rates, oocyte quality, and hormone levels vital for sheep reproduction (Sawyer and Narayan, 2019). Additionally, research suggests that heat stress disproportionately affects pregnant and lactating sheep compared to their non-pregnant or non-lactating counterparts (Belhadj Slimen et al., 2016).

Research on cattle also highlighted the negative impact of heat stress on follicle growth and function. In vivo heat stress for 20 to 26 days reduced steroid production from follicular cells in vitro (Maya-Soriano et al., 2013). Additionally, 12 hours of in vitro heat stress triggered early activation of primordial follicles, impaired oocyte nuclear maturation, and disrupted steroid production. The study conducted by Pöhland et al. (2020) has shown that when Cumulus-Oocyte Complexes (COC) is subjected to heat stress at 40 °C in vitro, the stress of ±1.5 °C compared with the ideal oocyte maturation temperature had negative effects on the cleavage and blastocyst rates but did not affect the rate of Polar Body (PB) extrusion in zebu cows. Hooper et al. (2015) reprorted that Cumulus-Oocyte Complexes (COCs) exposed to heat stress (41 °C) during the first 12 hours of IVM achieved metaphase II (MII) at rates comparable to non-stressed COCs (91.6% vs. 91.1%). However, heat-stressed oocytes experienced accelerated Germinal Vesicle Breakdown (GVBD), with most reaching GVBD within the first 6 hours of maturation.

The substantial heat generated during lactation in nursing animals complicates body temperature regulation during heat stress, exacerbating the effects on fertility in cows (Hansen, 2007). Furthermore, oocytes are highly responsive to elevated temperatures. Changes induced by heat in small antral follicles can lead to decreased maturation and reduced developmental capacity of the released oocyte. Rossi (2017) emphasizes the importance of the immediate follicular environment and explores potential mechanisms contributing to impaired oocytes. These mechanisms involve aspects related to nuclear and cytoplasmic maturation, mitochondrial function, pathways associated with cell death (apoptosis), and increased oxidative stress (Rana et al., 2014).

A recent study by Romo-Barron et al. (2019) examined the impact of heat stress on the reproductive capabilities of ewes. The research revealed that ewes exposed to thermoneutral conditions were significantly more likely to exhibit estrus, a period of sexual receptivity, compared to those subjected to heat stress. Heat stress has been found to harm the reproductive performance of cycling ewes. More specifically, research has revealed that heat stress reduces the duration of estrus by approximately 7.09 hours and extends the reproductive cycle by approximately 0.5 days. Furthermore, it has been demonstrated that ewes exposed to thermoneutral conditions are 2.4 times more likely to achieve pregnancy when compared to those exposed to heat stress (Romo-Barron et al., 2019).

**Figure 2:**
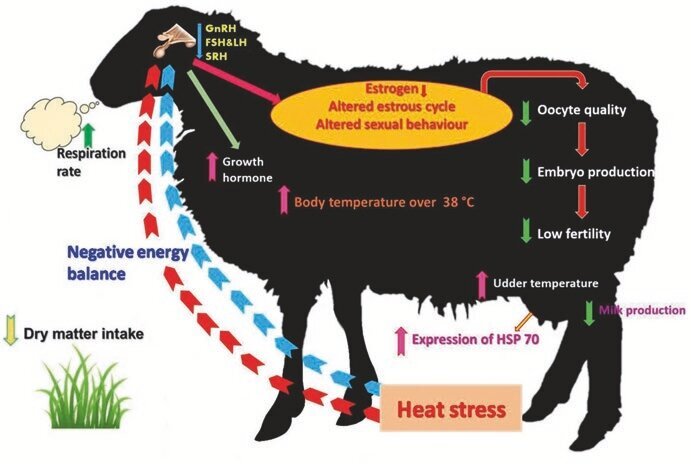
Effects of heat stress on reproductive activities in Ewe (Sejian et al., 2017a).

The adverse effects of heat stress were notably more pronounced in pregnant ewes when compared with those in the cycling phase. For instance, ewes that encountered heat stress during the initial trimester of gestation were about 12 times more prone to experiencing embryo mortality, with this risk escalating to approximately 26 times if the exposure to heat stress endured for a moderate period. While ewes exposed to heat stress from the second trimester of gestation did not exhibit effects on embryo mortality, reductions in both placental and fetal weights were observed, approximately by 180 grams and 1 to 1.6 kilograms, respectively (Romo-Barron et al., 2019). Moreover, it has been observed that elevated temperatures have detrimental effects on multiple aspects related to lambs, such as their birth weight, body weight gain, growth rate, total body solids, and daily solids gain. Extensive research has indicated that prolonged exposure of pregnant ewes to high environmental temperatures can have adverse effects on placental development and fetal growth, ultimately leading to an elevated risk of mortality or low birth weight among newborn lambs (Pérez et al., 2020). As shown in Figure 2.

## Heat stress effects on rams

The negative effects of elevated temperatures on male fertility are significant, potentially resulting in impairments in sperm production and motility, along with an elevation in the percentage of morphologically irregular sperm (van Wettere et al., 2021). In various mammalian species, including Humans, the testes are located in the scrotum external to the body (Zhu et al., 2004; Khan et al., 2020). This anatomical configuration plays a vital role in maintaining a slightly lower temperature in the testes compared to the central body temperature, thereby facilitating optimal reproductive functionality (Pineda and Dooley, 2003). This thermal gradient serves various physiological purposes, believed to facilitate efficient sperm production, reduce genetic mutations in germ cells, and support sperm maturation and storage within the epididymis (McManus et al., 2020).

The characteristics of ram semen, such as motility, volume, pH, concentration, and morphological abnormalities, are influenced shortly after exposure to high temperatures (Júnior et al., 2015). These changes typically manifest around two weeks after the onset of heat stress and persist for six to ten weeks after the stress subsides (Júnior et al., 2015). Breeds adapted to heat, particularly those with hair and wool, tend to recover more quickly compared to commercial wool and Dorper breeds. There were no significant differences in semen traits among these breeds before exposure to heat stress. Elevated testicular temperatures from overall body heat or specifically targeted at the testicles disrupt the spermatogenesis process, resulting in decreased sperm concentration, motility, normal sperm morphology, and reduced fertilization capacity (Zhu et al., 2004).

**Figure 3:**
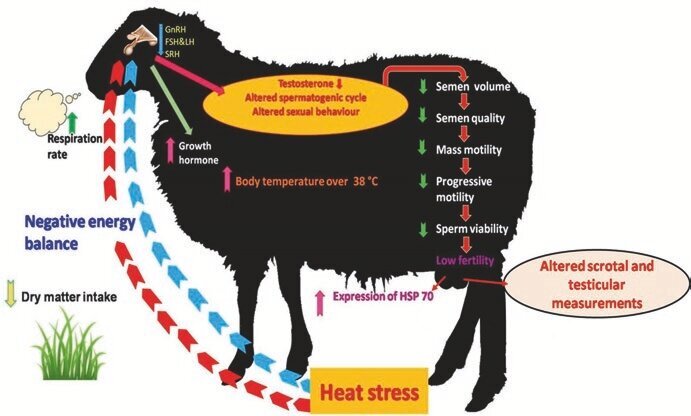
Effects of heat stress on reproduction in Ram (Kumar et al., 2018).

Moreover, thermal stress can have a direct impact by disrupting the reproductive pathways or an indirect impact by reducing feed intake to mitigate metabolic heat and induce an energy imbalance (Alves et al., 2016). Heat stress leads to a reduction in semen volume, decreased sperm motility, increased sperm defects, and decreased libido in both rams and bucks (Maurya et al., 2018). Overall, stress conditions result from heat influence sexual behavior, reducing sexual activity, lowering sperm quality, and decreasing the chances of conception. However, conflicting data exist regarding the level of serum testosterone in testes exposed to heat. Rasooli et al. (2010) noted no changes in serum testosterone concentration despite increased heat exposure, while other study suggested a decrease in testosterone levels in rams exposed to ambient temperature (Alves et al., 2016). As shown in Figure 3.

## Effects of heat stress on genes encoding reproductive function

Elevated temperatures have been shown to impact the expression genes that encode reproductive function in small ruminants (Mondal et al., 2017). This intricate process involves the synthesis of corticotropin-releasing hormone in the hypothalamus, triggering the secretion of Adrenocorticotropic Hormone (ACTH) from the pituitary gland (Sejian et al., 2012a). Afterward, Adrenocorticotropic Hormone (ACTH) induces the production of glucocorticoids and catecholamines, both of which have disruptive effects on genes encoding reproductive function. Herman et al. (2010) reported a decline in follicle-stimulating hormone (FSH) and its receptor gene (FSHR) expression in the pituitary glands of sheep subjected to heat stress. When environmental temperatures exceed 38°C, the expression of FSH—an essential hormone for ovarian follicle development—and its receptor gene (FSHR) within ovarian tissues decreases. This decline in Follicle-Stimulating Hormone Receptor (FSHR) gene expression hampers follicular growth, resulting in failed ovulation and causing sheep infertility (Wei et al., 2017). Intriguingly, Nyboe Andersen et al. (2017) showed in their study that heat-stressed ewes displayed increased expression of both Follicle-Stimulating Hormone (FSH) and Follicle-Stimulating Hormone Receptor (FSHR) genes upon administration of lipopolysaccharides. Regarding Luteinizing Hormone (LH), (Ozawa et al., 2005) noted a significant decrease in Luteinizing Hormone Receptor (LHR) gene expression in sheep experiencing heat stress. The reduction in LHR gene expression might be associated with reduced steroidogenic activity. Reduced estradiol levels during heat stress correlate with decreased androstenedione production in the theca cells, linked to limited expression of 17α-hydroxylase (Krishnan et al., 2017).

In sheep subjected to high temperatures, there is a marked upregulation in the expression of the *Prostaglandin F-2-alpha (PGF2α)* gene within the endometrium, possibly resulting from heat-induced changes in cellular membranes that augment the substrates available for prostaglandin synthesis (Mondal et al., 2017). This increase in Prostaglandin F-2-alpha (PGF2α) synthesis may stem from the heightened expression of the Cyclooxygenase-2 (COX2) gene in preimplantation embryo trophectoderm under heat stress conditions. The elevation in COX2 expression is influenced by increased HSF1 in heat-stressed animals, potentially contributing to the escalated levels of prostaglandin F-2 alpha. *FSHR, inhibin, LHR, GnRH, ESTR* (Estradiol receptor), and progesterone receptors are pivotal genes that have a significant impact on the regulation of reproductive function in small ruminants (Sheikh et al., 2017). Moreover, these genes as mentioned earlier can serve as valuable markers for evaluating reproductive efficiency in animals facing heat stress (Sheikh et al., 2017; Ahmad et al., 2021). Thermal stress impairs male fertility by directly affecting the reproductive axis or indirectly reducing feed intake to minimize metabolic heat production, leading to energy imbalance. It negatively influences male reproductive performance by decreasing semen volume and sperm motility, increasing sperm abnormalities, and lowering libido in both rams and bucks (Farshad et al., 2012). A recent study on Egyptian fat-tailed sheep identified several genes—*Connective Tissue Growth Factor (CTCFL), Microtubule-Associated Serine/Threonine-protein Kinase 2 (MAST2), Testis-Specific Protein Kinase 2 (TESK2), and initiator of meiotic double-strand break (SPO11)*—as potential candidates involved in male reproductive physiology (Mwacharo et al., 2017).

## Genes or candidate genes associated with heat stress in sheep

The initial stage in promoting adaptations to the environment within structured breeding programs entails identifying genomic regions linked to resistance against environmental stressors (Aslan et al., 2022). Over the past three decades, molecular techniques, including Polymerase Chain Reaction (PCR), have been extensively employed in animal genetics research. These methodologies encompass a range of approaches, such as Restriction Fragment Length Polymorphism, Amplified Fragment Length Polymorphism, microsatellites, and others (Aslan et al., 2022). Initially used for conservation initiatives, these methods concentrated on specific genetic loci throughout the genome to uncover Quantitative Trait Loci (QTLs) and enhance desired genotype combinations within populations through Marker-Assisted Selection (MAS) (Chen et al., 2015). Nonetheless, investigating a confined set of genetic locations associated with specific traits, like environmental adaptation influenced by multiple genes, presents a notable challenge in conservation and selection studies (Allendorf et al., 2010).

The rapid progress in Single Nucleotide Polymorphism (SNP) chips and sequencing of DNA technologies for species of livestock provides a new and crucial avenue to address these challenges. Whereas the exploration of QTLs using microsatellites typically revolves around the identification of a range of 30 to 100 loci, SNP arrays or Next-Generation Sequencing (NGS) Technologies can pinpoint 50,000 to 100,000 SNPs (Yan et al., 2020). This can be elucidated by the fact that equivalent genetic information can be derived from a single microsatellite and three SNPs (Fernández et al., 2013).

In contrast to microsatellites, which are conventional genetic markers, SNP arrays, and NGS technologies are widely recognized as reliable tools for genomic research due to their extensive genome coverage. The utilization of high-density genomic data derived from SNP arrays and NGS technologies is frequently employed to elucidate genomic regions that are associated with environmental adaptation (Cao et al., 2021). Genomic regions linked to environmental adaptation, which have been identified through the detection of selection signatures and Genome-wide Association Studies (GWAS), can be integrated into strategies for Genomic Selection (GS) (Sejian et al., 2019). These advancements in genotyping have significantly streamlined whole-genome inquiries in livestock and various scientific domains. Recent studies have identified candidate genes associated with high-altitude adaptation, climate change, and disease resistance in small ruminants. These genes were identified through genome analysis for selection signatures using single nucleotide polymorphism arrays and high-density genomic data obtained from next-generation sequencing Technologies (Fernández et al., 2013).

According to a study conducted by Garner et al. (2016), the utilization of genome-wide DNA markers has been shown to enhance the efficacy of genomic selection for various traits, such as heat stress tolerance. Similarly, Kim et al. (2016), identified specific candidate genes associated with heat stress in the Baraki desert sheep and goats in Egypt. Moreover, Yang et al. (2016) underscored the significance of Glutathione Peroxidase 3 (GPX3) in the arachidonic acid metabolism pathway, which plays a crucial role in the survival of sheep in desert environments (Table 1). Additionally, it has been documented that the presence of 11 regions on 12 chromosomes in Egyptian sheep exhibited potential selection sweeps, indicating their suitability for hot arid conditions. As shown in Table 1.

**Table 1:** Candidate genes associated with heat stress in sheep.

**Candidate genes**	**Function**	**Breed**	**Reference**
*Glutathione Peroxidase 3, Glutathione Peroxidase 7 and Prostaglandin Endoperoxide Synthase3*	Metabolism of arachidonic acid	Taklimakan desert sheep	(Gebreselassie et al., 2019)
*Carboxypeptidase A3, CarboxypeptidaseVitellogenic-Like, ECE1 and Endothelin-ConvertingEnzyme1*	Facilitate renin-angiotensin system	Desert Taklimakan sheep	(Gebreselassie et al., 2019)
*Solute Carrier Family 4 Member 4, Carboxypeptidase B1 and CPB1*	Signaling of Oxytocin	Desert Taklimakan desert sheep	(Gebreselassie et al., 2019)
*Mitochondrial Calcium Uptake 2, Intraflagellar Transport 88, and Oxytocin/vasopressin receptor,*	Secretion of Pancreatic tissue	Desert Taklimakan sheep	(Gebreselassie et al., 2019)
*UroporphyrinogenDecarboxylase,, Eukaryotic Translation Initiation Factor 2B, Polo-like Kinase 3 and Transglutaminase 3*	Heat stress/temperature stimuli	Baraki sheep	(Kim et al., 2016)
*Phospholipase C Beta 1, Fibroblast Growth Factor 2 and G Protein Subunit Alpha I3*	Heat-tolerance (melanogenesis)	Baraki goat and sheep	(Kim et al., 2016)
*Bone Morphogenetic Protein 4, Gap Junction Protein Alpha 3, and Bone Morphogenetic Protein 2*	Development and body size	Baraki goat and sheep	(Kim et al., 2016)
*Aldehyde Dehydrogenase 1 Family Member A3, Thyrotropin-Releasing Hormone Degrading Enzyme, and Myosin Heavy Chain,*	Digestive and energy metabolism	Baraki sheep and goat	(Kim et al., 2016)
*Interlukin7, Interleukin 21, Interleukin 1 Receptor Type 1, Interleukin 2 and Glutamate Ionotropic Receptor AMPA Type Subunit 1*	Autoimmune and nerve response	Baraki goat and sheep	(Kim et al., 2016)
*Transient Receptor Potential Cation Channel Subfamily M Member 8*	Body temperature regulation	Brazilian sheep	(de Simoni Gouveia et al., 2017)

## Genes related to sheep fertility

Fertility in sheep is a fundamental component in the economic dynamics of the sheep production sector. Key factors, including ovulation rate, litter size, and age at first parturition, significantly influence this aspect (Gebreselassie et al., 2019). Among these factors, ovulation rate and litter size hold particular importance. Although most sheep breeds typically give birth to a single offspring, certain breeds possess the genetic and environmental capacity to produce multiple offspring. Genetic factors play a crucial role in regulating fertility traits in sheep, with specific genes exerting influence on fecundity (Chen et al., 2015).

A Genome-Wide Association Study (GWAS) has yielded a comprehensive elucidation of the genes linked to litter size, ovulation rate, and sterility in various breeds of sheep. Prominent genes identified in this study include *Bone Morphogenetic Protein Receptor (BMPRIB*), *Bone Morphogenetic Protein (BMP15*) (Wang et al., 2015), *and Growth Differentiation Factor (GDF9)* (Hanrahan et al., 2004) (Table 2). *BMPRIB* primarily affects ovulation and litter size, while *BMP15 and GDF9* play pivotal roles in follicle development. *BMP15* specifically impacts granulosa cells, theca cells, and oocytes, whereas *GDF9* governs the progression of ovarian follicles. *GDF9* belongs to the *Transforming Growth Factor-beta (TGF-β)* family (Hanrahan et al., 2004; McNatty et al., 2005). The *FecB (Fecundity Boorola)* gene, was first discovered in highly prolific Booroola Merino sheep (Liu et al., 2014). These sheep carry a mutation (A746G) in the coding region of the *FecB* gene, resulting in the replacement of arginine with glutamine in the protein. The FecB gene influences ovulation rate additively and affects litter size, ranging from additive to dominant depending on the specific genotype (Fogarty, 2009). In addition to fertility traits, other economically significant factors include characteristics related to reproductive performance. For example, a study was carried out on particular sheep breeds utilizing microsatellite genotyping to detect genetic variations in the *Prolactin receptor (PRLR) gene* (Bodin et al., 2007). This gene is classified as a member of the class 1 cytokine receptors family and was determined to correlate with reproductive performance (Bowles et al., 2014).

**Table 2:** Genes related to fertility in sheep.

**Genes**	**Chromosome**	**Position (bp)**	**Traits**	**Sheep breed**	**Reference**
BMPRİB	6	29361947–29448079	ORS	Lacaune Sheep	(Bowles et al., 2014)
BMPRİB	7	29361947–29448079	LS	Han sheep	(Bowles et al., 2014)
BMP15	X	50970938–50977454	ORS	Cambridge & Belclare	(Wang et al., 2015)
BMP15	X	50970938–50977454	LS	Han sheep	(Wang et al., 2015)
CCNB2	7	48194193–48217973	OD	GMM, CMF & AWD	(Hanrahan et al., 2004)
GDF9	5	41841034–41843517	ORS	Cambridge & Belclare	(Hanrahan et al., 2004)
PRLR	16	38969273–39028126	RP	Herdwick & RFD	(Bodin et al., 2007)
SLC8A3	7	78697982–78837399	OD	GMM, CMF & AWD	(Chu et al., 2007)
TMEM154	17	4832841–4882002	İLV	Herdwick & RFD	(Chu et al., 2007)

Bp: Base pair; RP: Reproductive performance; ILV: Infection to the lentivirus; OD: Oocyte development; ORS: Ovulation rate & sterility; LS: Litter size; GMM: German mutton merino; AWD: African white Dorper; FD: Fells & Dalesbred; CMF: Chinese Mongolian fat.

Furthermore, a Genome-Wide Association Study (GWAS) has discovered genetic variations in genes such as *Cyclin B2 (CCNB2)* and *Solute Carrier Family 8 Member A3 (SLC8A3)* that are positively correlated with oocyte development in certain sheep breeds (Table 2). In particular, *SLC8A3* augments the availability of L-alanine and L-histidine for gap junctional transfer in oocytes. Another GWAS investigation has established a connection between the *Transmembrane protein 154 (TMEM154)* (Chu et al., 2007) gene and susceptibility to ovine lentivirus. This gene function reduces vulnerability to the lentivirus (Gebreselassie et al., 2019). As shown in Table 2.

## Conclusion

Heat stress poses a significant challenge to sheep reproduction, despite the species' ability to adapt to changing environments through alterations in physical and genetic characteristics. Sheep respond to heat stress by employing a range of adaptive mechanisms at the behavioral, morphological, physiological, and genetic levels. This review discussed these adaptive mechanisms and the detrimental impacts of heat stress on sheep reproduction, including reduced fertility manifested through effects on ovulation, estrus expression, conception rates, embryonic survival, fetal development, semen quality, changes in mating behavior, failed egg fertilization, post-mating loss of fertilized eggs, and fetal dwarfism. Furthermore, this present review critically assesses a range of genes, including candidate genes, that play a crucial role in offering protection against heat stress. For instance, the gene HSP70 is recognized for its significant cellular and molecular involvement in assessing sheep responses to heat stress. Genes responsible for encoding GnRH, FSHR, LHR, and ESTR may serve as indicators of the reproductive implications of heat stress in sheep. Cutting-edge molecular biology techniques such as whole transcriptome analysis and Next-Generation Sequencing (NGS) present promising opportunities for identifying enduring biological markers related to heat stress in sheep. Leveraging advanced molecular biology tools, targeting resilient genes, and conducting comprehensive investigations are imperative for comprehending the mechanisms through which small ruminants adapt to challenging environmental conditions.
